# Draft Genome Sequences of Two Multidrug-Resistant Salmonella enterica Serovar Typhimurium Clinical Isolates from Uruguay

**DOI:** 10.1128/MRA.00917-18

**Published:** 2018-08-02

**Authors:** Nicolás F. Cordeiro, Bruno D’Alessandro, Andrés Iriarte, Derek Pickard, Lucía Yim, José Alejandro Chabalgoity, Laura Betancor, Rafael Vignoli

**Affiliations:** a Departamento de Bacteriología y Virología, Instituto de Higiene, Facultad de Medicina, UDELAR, Montevideo, Uruguay; b Departamento de Desarrollo Biotecnológico, Instituto de Higiene, Facultad de Medicina, UDELAR, Montevideo, Uruguay; c The Wellcome Trust Sanger Institute, The Wellcome Trust Genome Campus, Hinxton, United Kingdom; Georgia Institute of Technology

## Abstract

Multidrug-resistant Salmonella enterica isolates are an increasing problem worldwide; nevertheless, the mechanisms responsible for such resistance are rarely well defined. Multidrug-resistant S. enterica serovar Typhimurium isolates ST3224 and ST827 were collected from two patients. The characteristics of both genomes and antimicrobial resistance genes were determined using next-generation sequencing.

## Abstract (precis)

Multidrug-resistant Salmonella enterica isolates are an increasing problem worldwide; nevertheless, the mechanisms responsible for such resistance are rarely well defined. Multidrug-resistant S. enterica serovar Typhimurium isolates ST3224 and ST827 were collected from two patients.

## ANNOUNCEMENT

Salmonella enterica is one of the main agents of foodborne diseases worldwide. This microorganism is responsible for nearly 90 million cases of human gastroenteritis annually, representing losses that reach €3 billion in Europe and $3.2 billion in the United States (see http://www.efsa.europa.eu/en/topics/topic/salmonella.htm and http://www.ers.usda.gov/data-products/cost-estimates-of-foodborne-illnesses.aspx#48446). Although antimicrobial treatment is not indicated for uncomplicated infection, fluoroquinolones, oxyimino cephalosporins, and azithromycin constitute the first line of treatment for extraintestinal S. enterica infection. The extensive use of such antimicrobials has led to the increase in antibiotic-resistant S. enterica isolates (1). We already reported the occurrence in Uruguay of extended-spectrum β-lactamases and plasmid-mediated quinolone genes in S. enterica isolates, with *bla*_CTX-M-14_ and *bla*_CTX-M-8_ being the most frequently detected (2, 3).

In the present study, two multidrug-resistant S. enterica serovar Typhimurium clinical isolates, ST3324 and ST827, were sequenced to analyze the genetic traits underlying their resistance phenotypes. The two strains were obtained in 2012 from fecal samples; ST3324 was isolated from an adult HIV patient, whereas ST827 was recovered from an elderly outpatient.

Genomic DNA was extracted using the Qiagen DNeasy blood and tissue kit. Whole-genome sequencing was performed using the Illumina HiSeq 2000 platform in 50- to 76-bp paired-end runs. High-quality reads were selected using Sickle (https://github.com/najoshi/sickle), and quality was assessed using FastQC (http://www.bioinformatics.babraham.ac.uk/projects/fastqc/). On average, 10,722,535 paired-end reads were produced for each strain, resulting in more than 105-fold coverage of each genome. *De novo* assembly was performed with SPAdes v. 3.6.1 (4) using a preassembly approach with Velvet v. 1.2.10 (5). The assembly was created using a range of k-mer sizes between 29 and 172 with a step of 2 and default parameters in both programs.

Totals of 191 and 183 contigs were generated for strains ST3224 and ST827, respectively, with a G+C content of approximately 52%. The genomes were annotated using the Rapid Annotations using Subsystem Technology (RAST) v. 2.0 server (6, 7); strain ST3224 featured 5,020 coding sequences (CDS) and 102 RNA-coding genes, whereas strain ST827 presented 5,063 CDS and 113 RNA-coding genes. Multilocus sequence typing (MLST) was conducted using MLST 2.0 (https://cge.cbs.dtu.dk/services/MLST/) (8), and both strains belonged to sequence type 19. Resistance genes were predicted with ResFinder 3.0 (9), and results indicated that strain ST3224 harbored two β-lactam resistance genes (*bla*_CTX-M-14_ and *bla*_TEM-1_), three aminoglycoside resistance genes [*aac(6′)-Iaa*, *aph(3′)-Ic*, and *aac(3)-IIa*], one quinolone resistance gene (*qnrB2*), one macrolide resistance gene (*ermB*), one tetracycline resistance gene [*tet*(A)], and three sulfonamide resistance genes (*dfrA25* and two copies of *sul1*). On the other hand, strain ST827 featured β-lactam resistance gene *bla*_CTX-M-8_, tetracycline resistance gene [*tet*(A)], sulfonamide resistance gene *sul2*, aminoglycoside resistance genes *aac(6′)-Iaa*, *strA*, and *strB*, and the phenicol resistance gene *floR*.

The occurrence of *Salmonella* isolates featuring resistance genes to the current therapeutic options calls for active surveillance policies to reverse this phenomenon.

### Data availability.

Sequence data of Uruguayan strains ST3224 and ST827 were deposited at the European Nucleotide Archive under accession numbers ERS354131 and ERS354132, respectively (BioSample numbers SAMEA2201728 and SAMEA2201729, respectively).
